# Single Neuron Optimization as a Basis for Accurate Biophysical Modeling: The Case of Cerebellar Granule Cells

**DOI:** 10.3389/fncel.2017.00071

**Published:** 2017-03-15

**Authors:** Stefano Masoli, Martina F. Rizza, Martina Sgritta, Werner Van Geit, Felix Schürmann, Egidio D'Angelo

**Affiliations:** ^1^Department of Brain and Behavioral Sciences, University of PaviaPavia, Italy; ^2^Dipartimento di Informatica, Sistemistica e Comunicazione, Università degli Studi di Milano-BicoccaMilan, Italy; ^3^Memory and Brain Research Center, Department of Neuroscience, Baylor College of MedicineHouston, TX, USA; ^4^Blue Brain Project, École Polytechnique Fédérale de LausanneGeneva, Switzerland; ^5^Brain Connectivity Center, C. Mondino National Neurological InstitutePavia, Italy

**Keywords:** granule cell, cerebellum, modeling, optimization techniques, intrinsic electroresponsiveness

## Abstract

In realistic neuronal modeling, once the ionic channel complement has been defined, the maximum ionic conductance (G_i-max_) values need to be tuned in order to match the firing pattern revealed by electrophysiological recordings. Recently, selection/mutation genetic algorithms have been proposed to efficiently and automatically tune these parameters. Nonetheless, since similar firing patterns can be achieved through different combinations of G_i-max_ values, it is not clear how well these algorithms approximate the corresponding properties of real cells. Here we have evaluated the issue by exploiting a unique opportunity offered by the cerebellar granule cell (GrC), which is electrotonically compact and has therefore allowed the direct experimental measurement of ionic currents. Previous models were constructed using empirical tuning of G_i-max_ values to match the original data set. Here, by using repetitive discharge patterns as a template, the optimization procedure yielded models that closely approximated the experimental G_i-max_ values. These models, in addition to repetitive firing, captured additional features, including inward rectification, near-threshold oscillations, and resonance, which were not used as features. Thus, parameter optimization using genetic algorithms provided an efficient modeling strategy for reconstructing the biophysical properties of neurons and for the subsequent reconstruction of large-scale neuronal network models.

## Introduction

Realistic modeling allows a faithful reconstruction of neuronal excitable properties based on the principles of neuronal biophysics (Koch, 1999; De Schutter, 2001). This approach requires a precise representation of the electrotonic structure of neurons and of their ionic membrane mechanisms through a variety of ionic channels. Thus, realistic modeling is, in essence, a modern expansion of the approach developed by Hodgkin and Huxley (1952) for the action potential (AP) in the squid giant axon. Despite the amount of parameters populating realistic models is huge, most of them are constrained by experimental measurements and the parameters that remain free are basically the maximum ionic conductances (G_i-max_). Experimentally, G_i-max_ can rarely be measured reliably due to space-clamp problems. Moreover, the ionic current identified by electrophysiological and pharmacological tools often reflects activation of a blend of different channel molecules rather than correspond to a single type of genetically identified channel. Thus, once the ionic conductances in a neuron have been identified and represented in a Hodgkin-Huxley-like style (HH), what is usually done is to empirically adjust their G_i-max_ until matching the neuronal firing pattern. This “*iterative multiparametric matching*” with large experimental datasets can lead to precise models (e.g., see D'Angelo et al., 2001; Solinas et al., 2007a,b; Diwakar et al., 2009; Subramaniyam et al., 2014; Masoli et al., 2015), but it is slow and laborious.

A recent technique that allows rapid and automatic parameter estimation is based on multi-objective evolutionary algorithms (MOEA), such as the “*Non-dominated Sorting Genetic Algorithms-II*” (NSGA-II; Deb et al., 2002) and “*Indicator-Based Evolutionary Algorithm*” (IBEA) (Zitzler and Künzli, 2004). These are based on a genetic approach, in which the unknown parameters are treated like the genes forming a chromosome (Deb et al., 2002; Zitzler and Künzli, 2004; Druckmann et al., 2007, 2008). To find the solutions, these algorithms require specific target parameters, for example “features” extracted from experimental traces. The features are used to define the basic properties of APs (such as amplitude and hyperpolarization depth), of neuron discharge (e.g., frequency and first-spike delay) and of subthreshold responses, in order to assess the fitness function. From the features one or multiple “objective” functions can be computed, that have to be minimized simultaneously and represent the “fitness” of the individuals. Each individual carrying a specific combination of parameters is part of a population. In each generation, the algorithm performs a ranking of the individuals of the population and removes a predefined number of the worst individuals. This makes room for new individuals derived from the retained individuals using genetic principles (e.g., cross-over, mutation, elitism). Retained and new individuals make up the next generation. While evolutionary algorithms can lead to fast approximation of neuronal firing patterns, some G_i-max_ combinations could be non-physiological (e.g., non-unique solutions). Therefore, a stringent test is required to assess whether, among the solutions provided by the optimization procedure, there are (at least) some that match the biological G_i-max_ distribution.

In order to face the issue, we need a neuron in which the G_i-max_ values have been precisely estimated in electrophysiological recordings providing stringent constraints to the mechanisms of AP generation. The GrC offers this unique opportunity. The GrC is one of the smallest neurons of the brain and this has allowed to achieve exceptionally good voltage-clamp conditions leading to a precise determination of ionic current gating kinetics and G_i-max_ values. These include the high-voltage activated Ca^2+^ current (Ca-HVA; Rossi et al., 1994), the Na^+^ current (Na, Nap, Nar) (Magistretti et al., 2006; Goldfarb et al., 2007; Dover et al., 2010), the inward rectifier K^+^ current (Kir) (Rossi et al., 1998, 2006), the A-type K^+^ current (KA), the voltage-dependent outward-rectifier K^+^ current (KV), and the K^+^ calcium dependent (KCa) (Bardoni and Belluzzi, 1994), the M-type slow K^+^ current (Kslow; D'Angelo et al., 2001). In addition, some models that have been previously developed using iterative multiparametric matching can be used for comparison (Gabbiani et al., 1994; D'Angelo et al., 2001; Nieus et al., 2006; Diwakar et al., 2009). Therefore, the question is whether advanced optimization procedures can capture the whole set of GrC properties through a set of maximum ionic G_i-max_ values compatible with those measured experimentally.

Here we show that an automatic parameter estimation procedure, the Optimizer Framework (OF) which is based on Druckmann et al. (2007, 2008, 2011) and improved to run with IBEA (Zitzler and Künzli, 2004), can indeed provide GrC models with a biologically plausible set of G_i-max_ values. These models can predict electroresponsive properties like inward rectification, near-threshold oscillations, theta-frequency resonance and AP conduction velocity that were not set as features. These results indicate that the OF generates biophysically accurate models endowed with appropriate ionic mechanism, providing the basis for reconstructing large-scale neuronal networks operating with arbitrary firing patterns.

## Methods

In this paper, a pipeline was developed to generate families of GrC mono-compartmental and multi-compartmental models, to optimize their G_i-max_ complement, and to validate the models through the simulation of electroresponsive properties not considered for model construction. The features used as templates were extracted from GrC spike discharges under the assumption that these contain all the information required to optimize G_i-max_ values. The present models can be defined “realistic” as far as they reflect a modeling strategy that implements neuronal membranes with biophysically-detailed mechanisms (see discussion in De Schutter, 2001; Santamaria et al., 2007; D'Angelo et al., 2016).

### Physiological data and feature extraction

*In vitro* patch-clamp recordings were performed from GrCs in acute cerebellar slices obtained from juvenile rats (postnatal day 21), as previously described (D'Angelo et al., 1998). The experiments reported in this paper were conducted according to the international guidelines from the European Union Directive 2010/63/EU on the ethical use of animals and approved by the local ethical committee of the University of Pavia, Italy.

The GrC showed typical electrophysiological properties consisting of regular firing in response to step current injection. Three different current steps (10, 16, and 22 pA) were used, which were deemed to appropriately represent the GrC discharge pattern. The experimental traces were then used as templates to define the features required for modeling. The features were extracted with eFEL (http://bluebrain.github.io/eFEL), an open source module for Python (Van Geit, 2015). Each *feature* was translated into a single *objective* and used to guide the OF (Deb et al., 2002; Zitzler and Künzli, 2004; Druckmann et al., 2007, 2008).

The *features* were chosen to parameterize typical aspects of GrCs electroresponsiveness. GrCs are silent at rest (with a resting membrane potential around −65 mV), and their subthreshold responsiveness is regulated by a fast inward rectifier K^+^ current. In the near-threshold region, a complex interaction between a persistent Na^+^ current and a slow M-like K^+^ current generates low-frequency oscillations. Following current injection, GrCs generate rapid APs showing relatively small amplitude in the soma and two phases of after-hyperpolarization (AHP) reflecting the intervention of a Ca^2+^-dependent K^+^ current and a slow voltage-dependent K^+^ current (D'Angelo et al., 1998, 2001). As defined in eFEL, the fast AHP depth was calculated as absolute voltage ad AHP depth, while the slow AHP depth was calculated as the minimum between two neighboring spikes (the first 5 ms excluded). The delay to initial discharge is tuned by an A-type current. The neuron generates regular high-frequency discharges and the firing frequency raises rapidly with current injection due to the high GrC input resistance. Accordingly, the features comprised resting membrane potential, AP width and height, fast and slow AHP depth, mean AP frequency and time-to-first spike, adaptation and coefficient of variation of the interspike interval (ISI-CV) (see Table 1). In aggregate, the features were carefully selected to match the fundamental parameters measured experimentally (documented in (D'Angelo et al., 1995, 1998). We therefore decided to use these as the minimal number of features that can provide a typical characterization of cerebellar granule cell spikes and firing. The features were considered for three different current injections. Each *objective* consisted of a single feature.

**Table 1:** **Features**.

	**10 pA**		**16 pA**		**22 pA**	
	**Exp**	**Models**	**Exp**	**Models**	**Exp**	**Models**
Resting voltage (mV)	−68.5 ± 12.5	−64 ± 0.5	−68.77 ± 11.68	−62.69 ± 0.44	−69.13 ± 11.67	−61.41 ± 0.5
AP height (mV)	20.93 ± 1.58	24.59 ± 9.22	19.25 ± 1.5	31.43 ± 1.61	17.7 ± 1.85	33.59 ± 1.32
AP width (mV)	0.67 ± 0.06	0.62 ± 0.02	0.69 ± 0.05	0.69 ± 0.02	0.71 ± 0.06	0.7 ± 0.02
AP half width (ms)	0.54 ± 0.07	0.49 ± 0.18	0.55 ± 0.067	0.51 ± 0.01	0.58 ± 0.07	0.49 ± 0.01
AHP depth (mV)	−59.21 ± 0.6	−63 ± 0.4	−58.3 ± 0.6	−62.69 ± 0.44	−57.19 ± 0.7	−61.41 ± 0.49
AHP depth slow (mV)	−52.69 ± 2.0	−50.96 ± 2.2	−48.93 ± 5.1	−55.56 ± 0.62	−32.7 ± 12.1	−50.28 ± 0.75
Time to first spike (ms)	31.9 ± 16.2	70.56 ± 25.97	19 ± 11.2	8.47 ± 3.67	14.65 ± 9.4	4.25 ± 2.78
Mean frequency (hz)	30 ± 16.2	12.795 ± 5.16	45 ± 21.2	56.95 ± 5.43	60 ± 39.4	95.09 ± 5.37
Adaptation index (ms)	0.1 ± 0.1	0.2 ± 0.3	0.3 ± 0.3	0.6 ± 0.2	0.3 ± 0.03	0.1 ± 0.05
ISI CV (ms)	0.2 ± 0.19	0.2 ± 0.2	0.2 ± 0.1	0.1 ± 0.3	0.2 ± 0.1	0.5 ± 0.1

*The table shows the mean values of features, obtained from experimental traces (Exp) using eFEL (Van Geit, 2015), and the corresponding values measured in the simulated traces obtained from optimized mono-compartment GrC models (these latter are reported as mean ± s.d. from 19 valid individuals). Note that simulated parameters fall close or around the mean features value*.

### Models construction and simulation

The GrC models were reconstructed using Python-NEURON scripts (Python 2.7; NEURON 7.3) (Hines et al., 2007, 2009). The models consisted of either one or multiple compartments generating morpho-electrical equivalents of the GrC. The voltage- and Ca^2+^-dependent mechanisms were distributed among the compartments when required (see Tables 2–**4**). With this approach, the models could reproduce GrC electroresponsiveness elicited by somatic current injection. Here we have reconstructed “canonical” GrC models, which simulate the most typical electrophysiological behavior of GrCs. The gating kinetics were taken from previous models, in which they have been normalized to 30°C and represented in HH style (D'Angelo et al., 2001) and subsequent upgrades (Nieus et al., 2006; Diwakar et al., 2009; Solinas et al., 2010). The Nernst equilibrium potentials were pre-calculated from ionic concentrations used in current-clamp recordings and maintained fixed, except for the Ca^2+^ equilibrium potential, which was updated during simulations according to the Goldman-Hodgkin-Katz equation. The maximum ionic conductances were the unknowns and their values were optimized in the OF (see below).

**Table 2:** **Ionic mechanisms in the mono-compartment GrC model**.

**Conductance/location**		**Range G_i-max_ (mS/cm^2^)**	**Erev (mV)**	**Description of channel**
Na	Soma	10.4–15.6	87.39	HH
Nap		1.60e-2 to 2.40e-2		
Nar		0.4–0.6		
KV		2.4–3.6	–84.69	
KA		3.2–4.8		
Kslow		0.28–0.42		
Kir		0.72–1.1		
KCa		3.2–4.8		
Ca-HVA		0.37–0.55	129.33	
Lkg1		4.54e-2 to 6.82e-2	–58	

*The table shows, for the different ionic channel types, the default conductance ranges and the specific reversal potential. The corresponding gating equations were written in HH style. The decay value of the Ca^2+^ concentration was β_Calc_ = 1.5/ms (for full description of the gating mechanisms refer to D'Angelo et al., 2001)*.

In each compartment, membrane voltage was obtained as the time integral of the equation (Yamada and Adams, 1989):

$$\begin{array}{l} \frac{dV}{dt}=-\frac{1}{C_{m}}\times \left\{\sum_{i}\left[{\text{g}}_{\text{i}}\times (\text{V}-{\text{V}}_{\text{i}})\right]+{\text{I}}_{\text{inj}}\right\} \end{array}$$

Where V is membrane potential, *C*_*m*_ membrane capacitance, g_i_ are ionic conductances and V_i_ reversal potentials (the subscript i indicates different channels), and I_inj_ is the injected current. Adjacent compartments communicated through an internal coupling resistance (Diwakar et al., 2009). The ionic conductances, g_i_, depend on G_i-max_ which are the OF unknowns, as well as on the kinetics of the gating particles for each individual channel, that are themselves functions of V and t. The whole mathematical description of the models and of the ionic channels is reported in previous papers (D'Angelo et al., 2001; Nieus et al., 2006; Diwakar et al., 2009; Solinas et al., 2010) and is not repeated here.

#### Model morphologies

In order to run the models in OF, special transformations to neuron morphology were needed, since in Neurolucida format (ASC) the soma has to be defined with a contour rather than a cylinder like in NEURON. Thus, a contour of the GrC soma with surface area equivalent to the original cylindrical compartment was created with a custom python script and added to the ASC file. For the mono-compartmental model, the equivalent spherical radius of 9.76 μm was used (D'Angelo et al., 2001). For the multi-compartmental model, the GrC morphology was derived from a previous model (Diwakar et al., 2009) and the equivalent spherical radius was 5.8 μm. As for the remaining compartments representing dendrites, initial segment an axon, the morphology was first exported from NEURON into NeuroML format (XML), and then into the final format using NLMorphologyConverter V0.9 (http://www.neuronland.org/NL.html).

In the multi-compartmental model, GrC morphology was simplified with respect to the previous model of (Diwakar et al., 2009). The dendrites were represented as four single compartments (15 μm length, 0.75 μm diameter). The axon initial segment (AIS) was represented as a single compartment (2.5 μm length, 1.5 μm diameter). The ascending axon was maintained unaltered with the same length (70 μm), diameter (0.3 μm), and number of segments but parallel fibers were not included. The original ion mechanisms were maintained unaltered and redistributed over the same (though simplified) model sections.

The granule cell is a very compact neuron with an electrotonic length *L* = 0.04 (Silver et al., 1992; D'Angelo et al., 1993), a value 2 orders of magnitude smaller than in neurons like Purkinje and pyramidal cells. Accordingly, the decay of membrane potential from soma to the end of a dendrite during an EPSP or a spike was shown to be <2% (D'Angelo et al., 1995). Therefore, dendritic branching is not an issue in terms of electrotonic decay, also considering that the dendrites are short (on average 13 μm; Hámori and Somogyi, 1983) and branches (at most consisting in a bifurcation) are uncommon. As far as compartmentalization is concerned, model reduction to a minimum effective number of compartments was tested beforehand (Diwakar et al., 2009). Again, the granule cells pose a very different situation from complex neurons (like pyramidal or Purkinje cells), for which much higher detail and fine-grain compartmentalization is needed to successfully account for complex dendritic branching and electrotonic properties. Therefore, more detailed reconstructions are not required in this context, in which we are testing the impact of ionic channel redistribution through compartments during the optimization process.

#### Model passive properties and active mechanisms

The granule cell passive properties were kept as in previous models (D'Angelo et al., 2001; Diwakar et al., 2009). Membrane capacitance (*C*_*m*_) was set at 1 μF/cm^2^, membrane resistance was determined by 1/G_tot_ Ω/cm^2^ (at rest the value is mostly determined by G_Leak_ and G_Kir_), axial resistance (R_a_) was set at 100 Ω^*^cm. The input Resistance (R_in_) calculated from current transients in voltage-clamp mode was 1.3 Ω in the monocompartmental model and 2.1 Ω in the multi compartmental model.

The mathematical reconstructions of ionic channels were those reported previously (see D'Angelo et al., 2001; Diwakar et al., 2009) and were kept unaltered in their kinetics and temperature to facilitate comparisons of results with previous models obtained using iterative multiparametric matching. Thus, the ionic channels of the mono-compartmental and multi-compartmental models were the same except for the Na^+^ channels. In the mono-compartmental model, three different representations were used for the resurgent (Na_r_), persistent (Na_p_), and transient (Na_t_) Na^+^ channels. In the multicompartmental model, Na^+^ channel gating was reproduced with a unified 13 state sodium channel (Raman and Bean, 2001; Khaliq et al., 2003; Magistretti et al., 2006).

#### Model simulations

Model simulations were performed on a 4-cores AMD FX 7500 CPU (8 GB ram) and on a single blade of a cluster, composed by 12 cores/24 threads (two Intel Xeon X5650 and 24 Gigabyte of DDR3 ram per blade). The simulations were all performed with variable time step (Hines and Carnevale, 2008) allowing to simulate the final population of models in <60 min. The results of each simulation was saved as a plain text file containing the time series of voltage as well as other relevant parameters (e.g., ionic current and Ca^2+^ concentration).

### Model optimization

**Figure 1:**
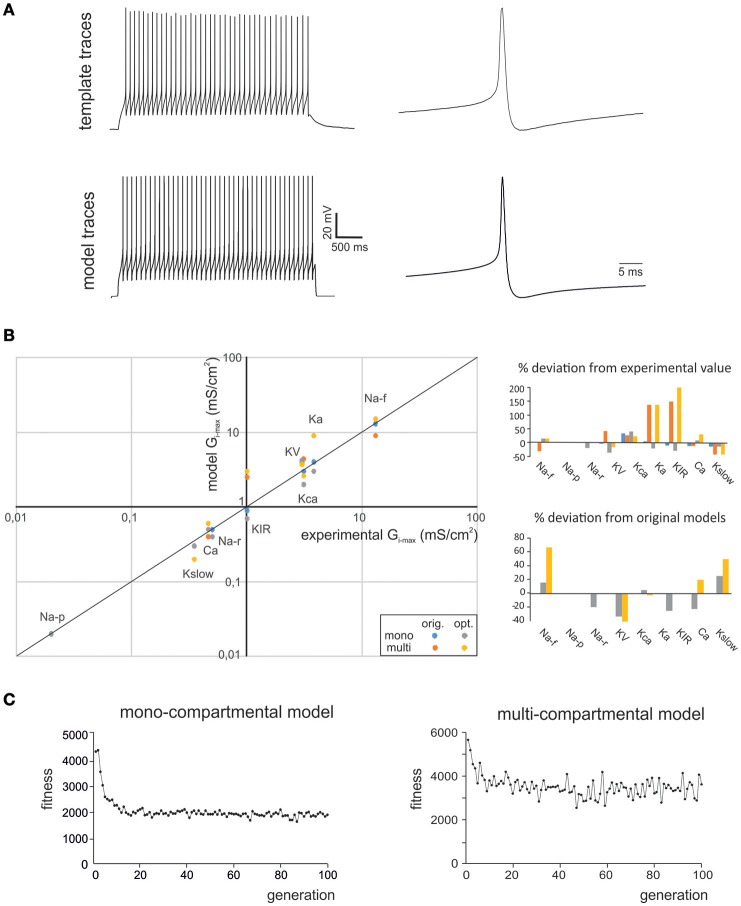
**Optimization of GrC firing pattern. (A)** The experimental data used for optimization were taken from GrC whole-cell current-clamp recordings (*top*). These data represented templates, from which the features describing AP shape and AP firing were extracted. The OF implemented a search in the parameter space that was able to find G_i-max_ combinations able to reproduce the experimental data. These G_i-max_ data-sets were used to reconstruct a population of individual GrC models and to simulate their AP and AP firing properties (*bottom*). The example shows experimental traces obtained with 10 pA current injection and their precise reproduction by a mono-compartmental GrC model. **(B)** The G_i-max_ values derived from experimental measurements: KA, KV, KCa: (Bardoni and Belluzzi, 1994); Na: (D'Angelo et al., 1998; Goldfarb et al., 2007); Ca-HVA: (Rossi et al., 1994); Kir: (Rossi et al., 2006); Kslow: (D'Angelo et al., 2001); are plotted against the G_i-max_ values obtained in the models (*left*). The values obtained using OF are the average obtained from all the individuals validated in the final generation (this paper). The values of the previous models are also reported (D'Angelo et al., 2001; Diwakar et al., 2009). For multicompartmental models, local ionic channel density was calculated from the experimental conductance values divided by the somato-dendritic surface in μm^2^. Note the strict correspondence of model and experimental values. The G_i-max_ deviation of model from experimental values and from optimized model form previous models is reported in the bar graphs (*right*). **(C)** The graphs show fitness evolution (in arbitrary units) during the optimization process. Note that either models required about 20 generation to halve the fitness attaining a non-0 steady state value.

The optimization procedure was performed using the IBEA genetic algorithm (Druckmann et al., 2007, 2008; Markram et al., 2015). The optimization procedure started from a default parameter range of G_i-max_ ± 50% derived from experimental measurements (see Figure 1B; KA, KV, KCa: Bardoni and Belluzzi, 1994; Na: D'Angelo et al., 1998; Goldfarb et al., 2007; Ca-HVA: Rossi et al., 1994; Kir: Rossi et al., 2006; Kslow: D'Angelo et al., 2001). Through a systematic variation of G_i-max_, OF produced populations formed by 150 individuals for the mono-compartmental or 200 individuals for the multi-compartmental models, respectively. During an iteration, the individuals were simulated and ranked by comparing the features extracted from the firing pattern of models to those of experimental templates in response to the same three positive current steps (10, 16, and 22 pA). The individuals best matching the prescribed features were automatically selected by an indicator function to seed the G_i-max_ parameter range of the next generation and so forth for 50 generations. This optimization cycle required 40 min for mono-compartmental and 90 min for multi-compartmental GrCs. The cycles were repeated 10 times. At the end of each cycle, the best individuals were selected and the range was reset to run the next cycle, and so forth. The *final population* was composed by the individuals of the last generation of the last cycle and was then fully simulated for validation (see results). In summary:

1. The initial range for parameter optimization was set based on the experimental G_i-max_ values (mean ± 50%) (see Figure 1B).
2. When OF was run, there was no supervision whatsoever while moving from one generation to the next, and parameter adjustment was automatically performed by OF.
3. Then, the Gi-max range was updated according to parameters estimated in these individuals and a new optimization cycle was started.
4. This process continued until the 10th cycle, in which the best individuals of the last generation were taken as the *final population* of models.
5. The individuals of the last generation were simulated and only those that generated spikes in response to all the three test current injections were considered. This criterion is more stringent than just ranking the best individuals since it selects only biologically valid solutions (indeed, the models that do not make spikes are not granule cells).

Documentation of the optimization algorithm used in the OF is available at http://www.tik.ee.ethz.ch/sop/pisa/selectors/ibea/ibea_documentation.txt and in the Zitzler paper (Zitzler and Künzli, 2004). We started by using the standard parameters provided by the simulation platform and, since there was a rapid convergence toward a stable solution and there were numerous models revealing a biophysically plausible parameter set (our target), we did not explore other parameter combinations. We used individual mutation probability = 0.5, individual recombination probability = 0.5, swap probability of 0.25. No elitism criteria were adopted. The slow convergence of genetic algorithms and the impact of stopping criteria are issues that have been clearly demonstrated previously for pyramidal neuron models (Druckmann et al., 2007). However, with granule cells, solutions converged rapidly and were almost at steady-state already after 20 iterations. After doing preliminary tests with 1,000 iterations, we decided to stop the optimization after 50 iterations (Figure 1C).

### Model validation

The validation procedure consisted in two simulation protocols. The first protocol repeated somatic current injections using 6 pA steps from 10 to 34 and 3 pA steps from −3 to −9 pA. This allowed to assess the voltage responses over both the negative and positive membrane potential range. The second protocol was a ZAP (Solinas et al., 2007a; Proddutur et al., 2013) used to determine the resonance properties of the neuron. The ZAP current injection was defined as A × sin (B × t^2^), where A = 10 pA is the oscillation amplitude and B (Hz/s) is a constant determining the oscillation speed. The input-output relationship was analyzed by calculating the interspike interval (ISI) and the instantaneous frequency for every couple of adjacent spikes. The average ISI per cycle was then plotted against the input frequency.

### Data analysis

Custom Python-NEURON scripts automatically transferred the set of parameters of the best individuals at the end of each cycle to the GrC model (either mono- or multi-compartmental), run the simulations and extracted the features. The voltage traces simulated using step current injections were passed to the same eFEL module used to analyze the experimental traces. Then the same features considered for the experimental traces were also extracted from model traces. The voltage traces simulated using ZAP current injections were analyzed using MATLAB scripts. Statistical analysis was performed with MS Excel obtaining mean ± *s.d*. of parameters.

### Comparison with experimental data

The values of the experimentally assessed ionic channel densities G_i-max_ were derived from published papers (Bardoni and Belluzzi, 1994; Rossi et al., 1994, 2006; D'Angelo et al., 1998, 2001; Goldfarb et al., 2007). These values data were compared to the homologous model parameter values (Figure 1B). In this figure local ionic channel density was calculated from the experimental conductance values divided by the somato-dendritic surface in μm^2^. Suppose for example that the same number of Na channels are localized either in the AIS or in the soma: this would result in different densities, since the corresponding surfaces are different. It should also be noted that it is unpractical to obtain experimental measurements of local ionic current densities in cellular compartments, except when these are physically isolated. This advanced procedure has been used in combination with patch-clamp recordings (both single-channel and whole-cell) and immunolabeling to determine the localization and density of Na currents in granule cells (Magistretti et al., 2006; Goldfarb et al., 2007; Dover et al., 2016). As a whole, the combination of data on channel subcellular localization and conductance densities provides a remarkable set of constraints for the present conductance-based realistic models.

## Results

In this work we used OF (Druckmann et al., 2007, 2008; Van Geit et al., 2016) to generate optimized GrC models that accounted for the ionic currents of real GrCs and to compare them to previous mono-compartmental [D'Angelo et al. (2001); updated in Nieus et al. (2006); Solinas et al. (2010)] and multi-compartmental GrC models [Diwakar et al. (2009); updated in Dover et al., 2016] tuned by iterative multiparametric matching. The features used here for optimization addressed GrC resting membrane potential, spike shape and firing pattern (Table 1). The matching of optimized model responses with the template is illustrated in Figure 1A. The correspondence of G_i-max_ in the optimized models with those measured experimentally in GrCs is shown in Figure 1B, along with a comparison of parameters in the optimized models with respect to previous models. Strikingly, the experimental and modeled G_i-max_ values (both for the optimized and previous models) showed an almost linear correspondence across 4 orders of magnitude, ranging from about 10^−2^ to 10^2^ mS/cm^2^. This precise correspondence implied that in most cases model parameters differed from the experimental ones by <20%, and the same was true for the difference between model parameters obtained using OF or procedures of iterative multiparametric matching. The largest variations were observed for the multicompartmental models, probably reflecting an imperfect knowledge about the localization of some ionic channels. The solutions converged rapidly and were almost at steady-state already after about 20 iterations for either models (Figure 1C). It should be noted that the non-0 steady-state level demonstrated a continuous remixing of genes over the entire population and the maintenance of genetic variability.

### Optimization of the mono-compartment GrC model

**Figure 2:**
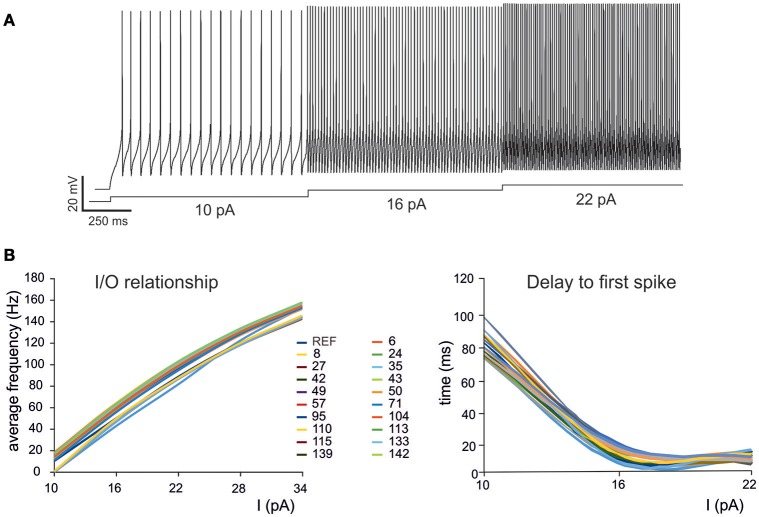
**Electroresponsive properties of a GrC mono-compartmental model. (A)** Simulation of AP firing of a GrC model selected among the individuals composing the final population. The simulation consisted of three current injections of 10, 16, and 22 pA lasting a total of 5 s. **(B)** Frequency/intensity relationships and delay to first spike at different current injections (10, 16, 22 pA). A previous model (D'Angelo et al., 2001) is compared to 19 GrC models resulting from OF (*left*). Note the close similarity between the original data-set and the OF models.

**Figure 3:**
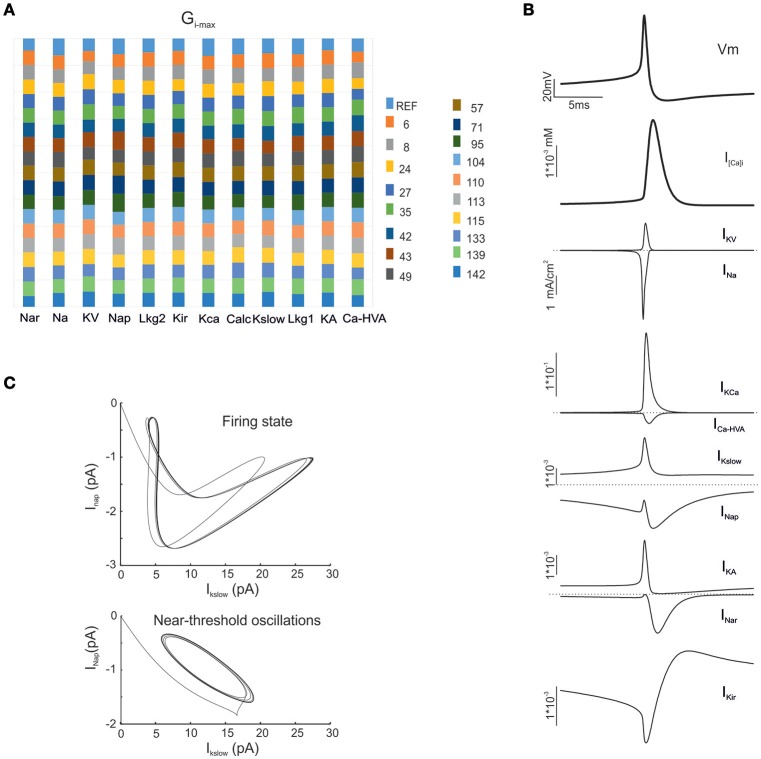
**Electroresponsive mechanisms in mono-compartmental models. (A)** The conductance value of each ionic channel was normalized and reported in columns for the valid model, allowing the comparison among individuals. Each channel type is defined by its name, except for “Calc” which was used to indicate the decay value of the Ca^2+^ concentration (D'Angelo et al., 2001). **(B)** Membrane voltage, Ca^2+^ concentration and ionic currents generated by an individual model. **(C)** Phase plots of the interaction between I_Kslow_ and I_Nap_ currents during firing *(top)* and during subthreshold oscillations *(bottom)*.

As a first step, we optimized a mono-compartment GrC model using the ionic current mechanisms and passive properties reported previously (D'Angelo et al., 2001); updated in Nieus et al. (2006) and Solinas et al. (2010). The OF produced models ranging from those non-responsive to any current injections, to others showing either irregular firing, responses to low injected currents only or proper GrC firing. Among all the individuals of the final generation, only a limited number (12.7%) generated appropriate resting membrane potential, spike shape and firing pattern (Figures 1A,B). In particular, these models had resting membrane potential around −65 mV, were silent at rest and generated regular firing above about 10 pA current injection (Figure 2A). By increasing the injected current, firing increased with a slope f/I = 7 ± 1 spikes/pA and spike delays decreased from about 100 to 1 ms, showing therefore the typical behavior of real GrCs (Figure 2B) (cf. D'Angelo et al., 1995, 1998; Brickley et al., 1996; Cathala et al., 2003). In these models, the distance of G_i-max_ from experimental values estimated previously was lower than 12.3% (Figure 3A). Moreover, intracellular Ca^2+^ and ionic currents underlying spike generation (Figure 3B) were strictly corresponding to those obtained through direct parameterization of G_i-max_ on the experimental data (D'Angelo et al., 2001). Likewise, the dynamics of two specific currents, I_Nap_ and I_Kslow_, generated a close cycle both in the near-threshold and supra-threshold regime (Figure 3C), providing the basis for low-frequency oscillations (D'Angelo et al., 2001).

**Figure 4:**
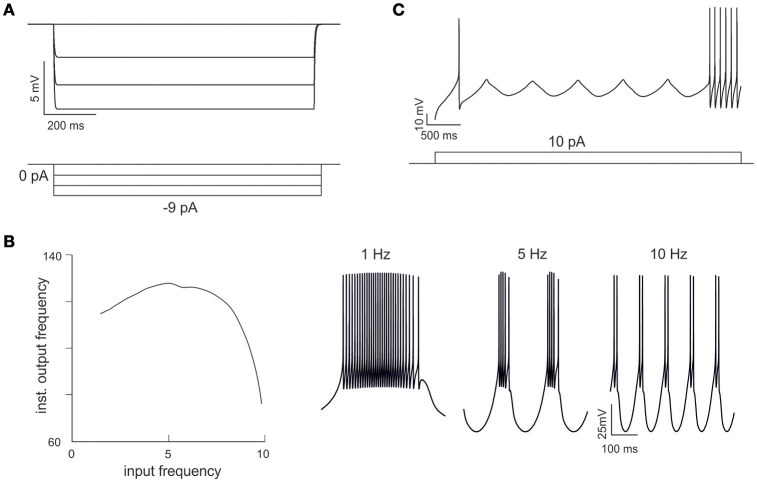
**Emerging properties of mono-compartmental models. (A)** A series of negative current pulses reveals the emergence of inward rectification. **(B)** Sinusoidal current injection (10 pA from rest from 0 to 10 Hz) reveals the presence of theta-frequency resonance. The plot shows peak response frequency around 4.5 Hz. The traces on the right (1, 5, and 10 Hz) illustrate the enhancement in instantaneous frequency at the resonance peak. **(C)** Step current injection reveals the emergence of oscillations in the near-threshold region.

Interestingly, in order to optimize G_i-max_ values, we used features extracted from GrC discharge in response to three depolarizing current pulses of increasing intensity. In theory, the information needed to parameterize the whole set of G_i-max_ values is all contained in these template traces, but in practice it may be difficult to extract appropriate parameters for those response regimens that are not explicitly represented in the templates. Nonetheless, OF was able to predict not just resting membrane potential, f/I relationship, first-spike delay and spike shape, but also *inward rectification* (Figure 4A), *resonance* (Figure 4B) *and near-threshold oscillations* (Figure 4C) in the appropriate frequency range (4–6 Hz). These were observed in all the neurons accepted on the basis of comparison with templates and can be considered as emerging properties deriving from the biophysical plausibility of model mechanisms. Therefore, the voltage traces elicited by current injection contained enough information to fully recover the fundamental electroresponsive properties of the neuron as a whole.

### Optimization of the multi-compartment GrC model

As a second step, we optimized a multi-compartment GrC model. This was a simplified version of the (Diwakar et al., 2009) model, in which the number of compartments in dendrites and axon was purposefully reduced in order to facilitate control over ionic channel distribution. The only additional constraint imposed to the model was channel localization, that was derived from previous experimental observations, so that Na^+^ channels were placed in the AIS and axon (Magistretti et al., 2006; Goldfarb et al., 2007) but not in soma or dendrites. The remaining ionic channels were distributed between soma and dendrites (Tables 3, 4). Therefore, the OF had to find solutions balancing G_i-max_ values of Na^+^, Ca^2+^, and K^+^ channels among dendrites, soma, AIS, and axon.

**Table 3:** **Electrotonic compartments in the multi-compartment GrC model**.

**Section name**	**Diameter (μm)**	**Length (μm)**	**No. of sections**	**Specific cm (uF/cm^2^)**
Dendrites	0.75	15	4	1
Soma	5.8	5.6	1	
AIS	1.5	2.5	1	
Axon	0.3	70	1	

*The table shows the sections of the multi-compartment GrC model along with their number, diameter and length*.

**Table 4:** **Ionic mechanisms in the multi-compartment GrC model**.

**Conductance/location**		**Range G_i-max_ (mS/cm^2^)**	**Erev (mV)**	**Description of channel**
Na	AIS	179.1–388.6	87.39	Markovian
	Axon	1.74–2.9		
KV	AIS	26.7–44.5	−84.69	HH
	Axon	3.3–5.59		
KA	Soma	4–10		
Kslow	Soma	0.18–0.31		
Kir	Soma	1.91–3.18		
KCa	Dendrites	2.85–4.76		
Ca-HVA	Dendrites	4.38–14.3	129.33	
Leak	Dendrites	1.6908e-2 to 2.4798e-2	–16.5	
	Soma	8.04e-2 to 0.13		
	AIS	7.214e-2 to 0.25		
	Axon	6.4319e-3 to 1.071e-2		

*The table defines the ionic channels, their location and the default range for each channel and the reversal potential. The corresponding gating equations were written in HH style. The decay value of the Ca^2+^ concentration was β_Calc_ = 1.5/ms for soma and β_Calc_ = 0.6/ms for dendrites (for full description of the gating mechanisms refer to Diwakar et al., 2009)*.

**Figure 5:**
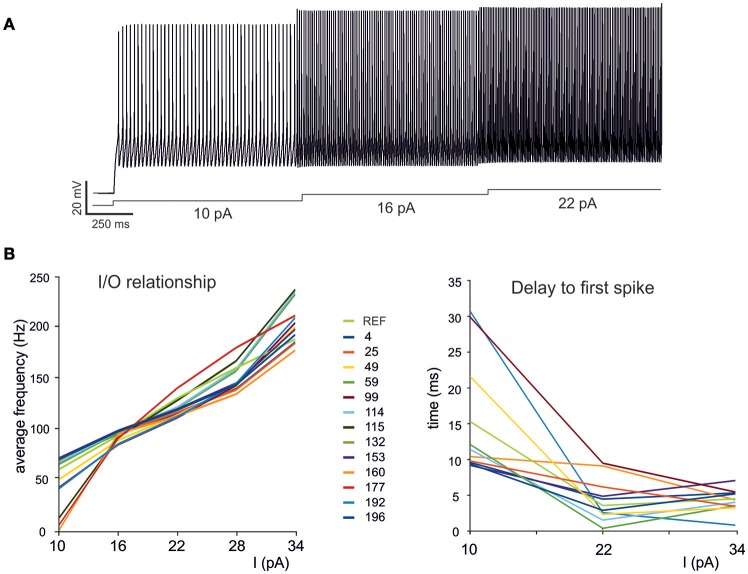
**Electroresponsive properties of a GrC multi-compartmental model. (A)** Simulation of AP firing of a GrC model selected among the individuals composing the final population. The simulation consisted of three current injections of 10,16, and 22 pA lasting a total of 5 s. **(B)** Frequency/intensity relationships and delay to first spike at different current injections (10, 16, 22 pA). A previous model (Diwakar et al., 2009) is compared to 13 GrC models resulting from OF (*left*). Note the close similarity between the original data-set and the OF models.

**Figure 6:**
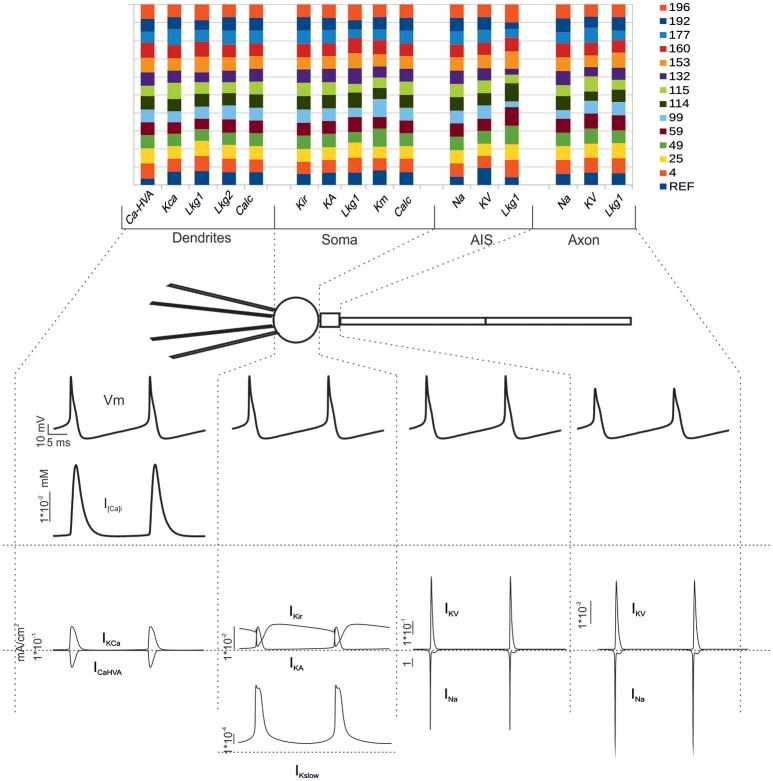
**Electroresponsive mechanisms in multi-compartmental models. (Top)** The conductance value of each ionic channel was normalized and reported in columns for valid models, allowing the comparison among individuals. Each channel type is defined by its name, except for “Calc” which was used to indicate the decay value of the Ca^2+^ concentration (Diwakar et al., 2009). **(Bottom)** Membrane voltage, Ca^2+^ concentration and ionic currents generated by an individual model. G_i-max_ were divided based on the actual distribution in the dendrites, soma, AIS and axon. The bottom traces show membrane voltage, Ca^2+^ concentration and ionic currents in the different sections.

**Figure 7:**
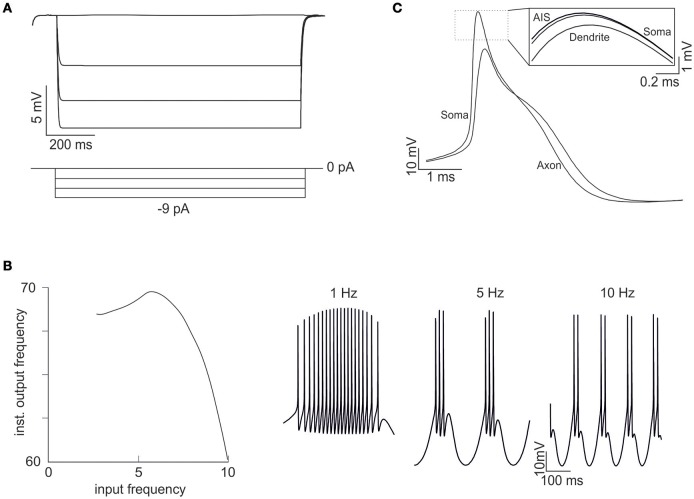
**Emerging properties of multi-compartmental models. (A)** A series of negative current pulses reveals the emergence of inward rectification. **(B)** Sinusoidal current injection (10 pA from rest from 0 to 10 Hz) reveals the presence of theta-frequency resonance. The plot shows a peak response frequency around 4.5 Hz. The traces on the right (1, 5, and 10 Hz) illustrate the enhancement in instantaneous frequency at the resonance peak. **(C)** Spikes in the soma and axon. The inset shows that the spike is generated first in the AIS and then back-propagates into the soma and dendrites with sub-millisecond delay. The distance between the soma and the middle of the axon is 45 μm and the conduction speed is 0.23 ms/mm.

Also in this case, as with the mono-compartmental model, the OF was able to find GrC models (6.5%) generating proper firing patterns, resting membrane potential and spike shape (Figure 5A). In particular, starting from a resting membrane potential around −76 mV, these models showed appropriate firing rates (mean 12 ± 6 spikes/s) and ranges of first-spike delays (8–30 ms; mean 15 ± 8 ms) (Figure 5B). All these models showed a similar balance among their G_i-max_ values and appropriate activation of ionic currents (Figure 6). As well as the mono-compartmental model, in these multi-compartmental models there were inward rectification and resonance (Figures 7A,B), while near-threshold oscillations were not evident. In general, the spike generation process was more explosive in the multi-compartment than in the mono-compartment models, a fact that could be due to the incomplete description of the AIS and axon structure and mechanisms (see Diwakar et al., 2009; Dover et al., 2016). Importantly, the spikes arose in the AIS, traveled at the proper speed (0.2 mm/ms) in the axon and back propagated rapidly (0.2 ms) in the dendrites (Figure 7C).

## Discussion

This paper shows that feature extraction from firing pattern templates combined with IBEA optimization/selection methods (OF; Druckmann et al., 2007, 2008) allow to generate neuronal models with ionic mechanisms that closely reflect those of real cerebellar GrCs (cf. Figure 1). These models are capable of uncovering properties that were not evident in the templates and derive uniquely from the appropriateness of the whole set of G_i-max_ values. To our knowledge, this is amongst the first OF applications to neurons other than those of the neocortex and hippocampus (for a recent application see Eyal et al., 2016).

The use of OF to parameterize cerebellar GrC models, for which a previous experimental determination of G_max−i_ is available (D'Angelo et al., 2001; Diwakar et al., 2009), illustrates some general aspects of the procedure. First, firing patterns recorded from the soma were sufficient to provide the information needed to fully parameterize the whole set of G_i-max_ values. The average G_i-max_ fluctuation was 12.3% for the mono-compartment models and 19.7% for the multi-compartment models. In both cases, most channels G_i-max_ fluctuations are between the variation limits suggested as acceptable for realistic modeling (≤ ± 20%, Bower and Beeman, 2007). Secondly, the OF neuron models proved correct against a set of properties used as features, including the spike shape and the dependence of firing frequency and first-spike delay on the injected current. This is akin with the empirical practice of matching features by adjusting model parameters by trial-and-error iterations and parametric fitting (e.g., Solinas et al., 2007a,b; Subramaniyam et al., 2014; Masoli et al., 2015). Thirdly, the model predicted properties not represented in the firing pattern and not used as features, including inward rectification, resonance and near-threshold oscillations. This is a proof of the plausibility of the underlying biophysical mechanisms, that allowed the whole set of neuronal properties to emerge. Consistently, G_i-max_ and membrane currents were similar to those recorded experimentally. Of particular relevance are the size and temporal dynamics of small ionic currents, that do not contribute remarkably to the total current (I_Nap_ and I_Kslow_ are 3 orders of magnitude smaller than the I_Na_ and I_KV_ currents) but were non-etheless matched by OF. Finally, with the GrC multi-compartmental model, indications about ionic channel localization had to be anticipated in order to allow OF to appropriately reconstruct the spike generation mechanism. This was an essential constraint, otherwise the models may still be able to generate a seemingly good firing pattern but the underlying mechanism would be incorrect (for example Na^+^ channels may well-generate spikes while staying in the soma, data not shown).

Beside the effectiveness of this optimization approach, there are some aspects that deserve attention. The identification of models with plausible biophysical properties required a validation going beyond the matching of features characterizing the firing pattern. For example, for the GrC models to be valid, the presence of resonance and near-threshold oscillation needs to be ascertained even if it is not included into the features. Therefore, following a selection process based on quality indicators (Sutskever et al., 2015), the final validation can further limit realistic models to a subset of all optimized models. Validation proved critical for both the multi-compartment and the mono-compartment models, with the percentage of neurons showing a plausible firing and spike shape being 13 and 6.5%, respectively. The rendering and requirements for validation in very complex neuron models (e.g., in the pyramidal neuron; Traub et al., 1991; Druckmann et al., 2011) or in the Purkinje cell neuron models (De Schutter and Bower, 1994a,b; Achard and De Schutter, 2006; Masoli et al., 2015) needs to be evaluated.

Given that all granule cells normally show stereotyped firing patterns under a unified mechanism, the fact that not all models in the final population do the same raises a relevant issue. Is it possible that, in biology, specific mechanisms constrain the solution toward the ionic channel asset needed to reach a specific firing pattern? These mechanisms may reside in yet undiscovered feed-back biochemical processes regulating G_i-max_ values through channel expression or modulation. Moreover, the fact that we have accepted only those solutions conforming to the most typical or “canonical” description of GrCs might have restricted the acceptance criteria. The OF may actually be able to predict model variants, whose correspondence with existing biological states is currently unclear and requires experimental assessment.

Special consideration deserves the AP generating process. The careful investigation of GrC electrophysiology has revealed that Na^+^ channels are almost absent from soma and are maximally concentrated in the AIS (Magistretti et al., 2006; Goldfarb et al., 2007). In the present model, AP passive back propagation to soma and dendrites occurred with proper delay and the active propagation in the axon occurred at the proper speed. These can again be considered as emerging properties depending on model structure and ionic channel distribution and density. A very recent study showed that the GrC axon contains Na^+^ channels that have different gating kinetics from those of the AIS and that the specific axon leak resistance is several times higher than in AIS, soma and dendrites (Dover et al., 2016). This allows the axon to lower by several time the energy consumption compared to the classical HH mechanism. These special properties of the axon, that have not been considered here, may be used as further constraints in the optimization process.

One may speculate on the way the optimization algorithm predicted the emerging phenomena. Probably, as far as inward rectification is concerned, the Kir conductance was set by extracting information from the current needed to move from rest to AP threshold and from the anomalous slow-down in first spike delay as the current injection was increased. And since the same feature is also controlled by KA, this could have generated some uncertainty in the conjoint estimation of these two parameters. Likewise, the I_Nap_/I_Kslow_ balance was probably determined by setting the firing threshold and the subsequent f/I relationship. Therefore, given representative templates of the firing patterns, the OF could predict the cell response in the subthreshold and near-threshold functional regimes, that were not considered as features. The same consideration applies to resonance, which depends on I_Kslow_ and is amplified by I_Nap_.

In conclusion, the OF using IBEA provided an objective automatic strategy for reconstructing the biophysical properties of neurons through a realistic set of ionic currents, reducing optimization times by orders of magnitude compared to traditional operator-guided procedures (days vs. months or years). The OF-derived models required a validation accounting for response patterns not used for model construction. This validation, by being itself based on biological constraints, does not introduce any arbitrary selections. The unsupervised optimization through OF confirmed the precision of multi-parametric matching procedures used previously for the same neurons. The ability of the OF models to respond to low-frequency oscillations and bursts of various frequency and duration makes them suitable for reconstructing large-scale models of neuronal microcircuits.

## Author contributions

SM wrote the model codes and the performed most of the simulations with the contribution of MR. MS performed the experiments. WV and FS supported model implementation and the discussion of principles. ED coordinated the work and wrote the paper with the contribution of all other Authors.

### Conflict of interest statement

The authors declare that the research was conducted in the absence of any commercial or financial relationships that could be construed as a potential conflict of interest.
